# The SARS-CoV-2 Spike Glycoprotein Biosynthesis, Structure, Function, and Antigenicity: Implications for the Design of Spike-Based Vaccine Immunogens

**DOI:** 10.3389/fimmu.2020.576622

**Published:** 2020-10-07

**Authors:** Liangwei Duan, Qianqian Zheng, Hongxia Zhang, Yuna Niu, Yunwei Lou, Hui Wang

**Affiliations:** ^1^ Henan Key Laboratory of Immunology and Targeted Drugs, School of Laboratory Medicine, Xinxiang Medical University, Xinxiang, China; ^2^ Henan Collaborative Innovation Center of Molecular Diagnosis and Laboratory Medicine, Xinxiang Medical University, Xinxiang, China

**Keywords:** SARS-CoV-2, spike glycoprotein, receptor-binding domain, synthesis, structure, membrane fusion, neutralizing antibodies, immunogen design

## Abstract

The ongoing pandemic of coronavirus disease 2019 (COVID-19), caused by severe acute respiratory syndrome coronavirus 2 (SARS-CoV-2), poses a grave threat to global public health and imposes a severe burden on the entire human society. Like other coronaviruses, the SARS-CoV-2 genome encodes spike (S) glycoproteins, which protrude from the surface of mature virions. The S glycoprotein plays essential roles in virus attachment, fusion and entry into the host cell. Surface location of the S glycoprotein renders it a direct target for host immune responses, making it the main target of neutralizing antibodies. In the light of its crucial roles in viral infection and adaptive immunity, the S protein is the focus of most vaccine strategies as well as therapeutic interventions. In this review, we highlight and describe the recent progress that has been made in the biosynthesis, structure, function, and antigenicity of the SARS-CoV-2 S glycoprotein, aiming to provide valuable insights into the design and development of the S protein-based vaccines as well as therapeutics.

## Introduction

The coronavirus disease 2019 (COVID-19) global pandemic represents an unprecedented public health, social and economic challenge (1, 2). The etiological agent of COVID-19 is a new member of the Coronaviridae family that is closely related to severe acute respiratory syndrome coronavirus (SARS-CoV) and was recently referred to as SARS-CoV-2 by the Coronavirus Study Group of the International Committee on Taxonomy of Viruses (3). The virus has spread rapidly and sustainably around the global resulting in over twenty-one million cases and more than 750,000 deaths as of August 15, 2020 (4).

Coronaviruses (CoVs) are enveloped positive-sense RNA viruses (5). Enveloped CoVs entering host cells and initiating infection is achieved through the fusion of viral and cellular membranes (6, 7). Membrane fusion is mediated by the large type I transmembrane S glycoprotein on the viral envelope and the cognate receptor on the surface of host cells (8–10). The surface-exposed location of the S glycoprotein not only allows it to carry out membrane fusion but also renders it a direct target for host immune responses, making it the major target of neutralizing antibodies (11). Because of its central roles in viral infection and eliciting protective humoral and cell-mediated immune responses in hosts during infection (10), the S protein is the primary target for vaccine design as well as antiviral therapeutics (12).

Here, we provide a comprehensive overview of the wealth of research related to the SARS-CoV-2 S glycoprotein biosynthesis, structure, function, and antigenicity, aiming to provide useful insights into the design and development of the S protein-based vaccines as well as therapeutics to prevent or treat the ongoing global spread of SARS-CoV-2/COVID-19.

## Synthesis, Processing and Trafficking of the SARS-CoV-2 S Glycoprotein

**Figure 1:**
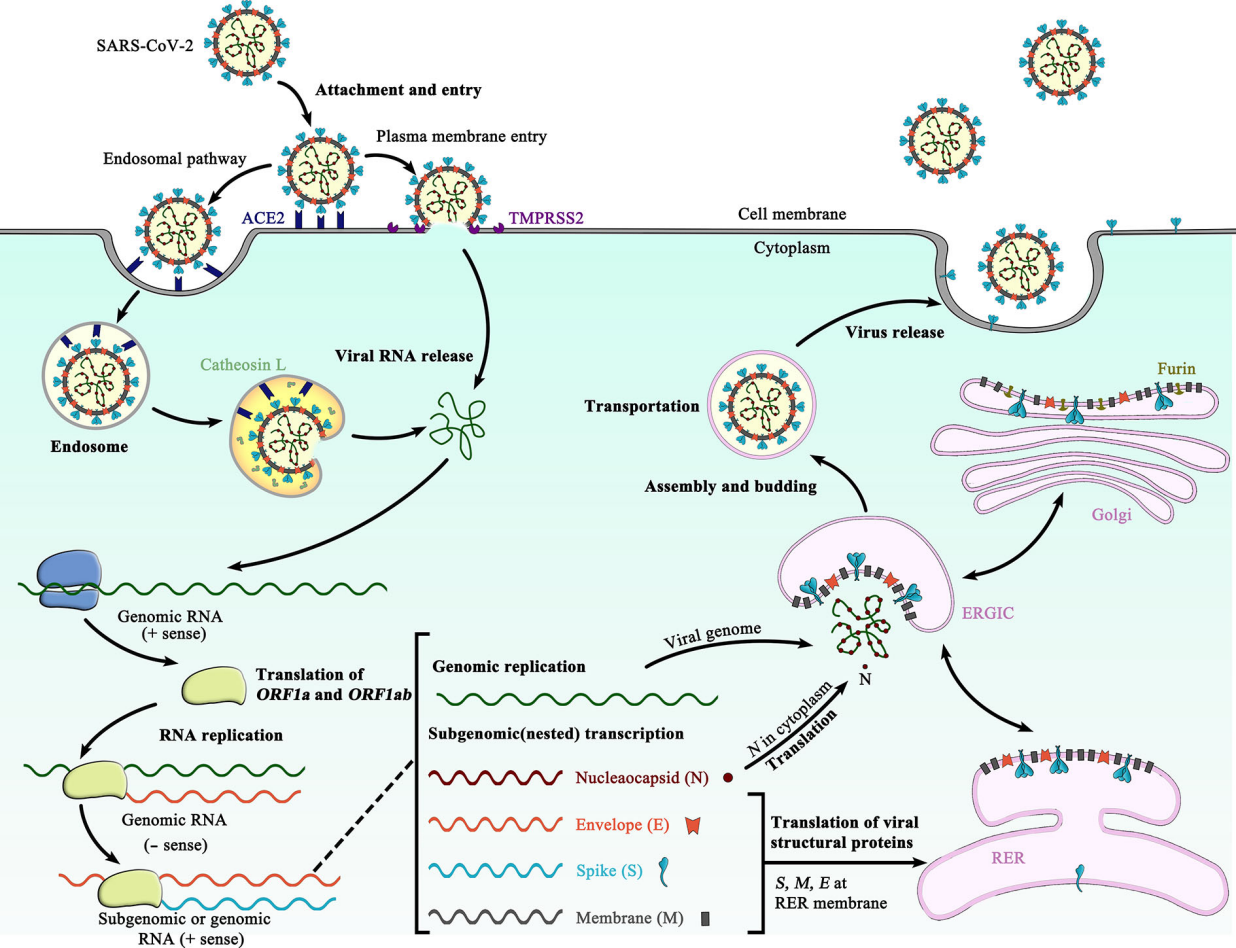
Schematic representation of the life cycle of SARS-CoV-2. The life cycle of SARS-CoV-2 begins with membrane fusion occurring at the plasma membrane or within acidified endosomes after endocytosis, which is mediated by conformational changes in the S glycoprotein triggered by angiotensin-converting enzyme 2 (ACE2) binding. Following viral entry, SARS-CoV-2 releases its genomic RNA into the host cell cytoplasm. Genome RNA is first translated into viral replicase polyproteins (pp1a and 1ab), which are further cleaved by viral proteases into a total of 16 nonstructural proteins. A replication-transcription complex (RTC) is formed based on many of these nonstructural proteins. In the process of genome replication and transcription mediated by RTC, the negative-sense (− sense) genomic RNA is synthesized and used as a template to produce positive-sense (+ sense) genomic RNA and subgenomic RNAs. The nucleocapsid (N) structural protein and viral RNA are replicated, transcribed, and synthesized in the cytoplasm, whereas other viral structural proteins, including the S protein, membrane (M) protein and envelope (E) protein, are transcribed and then translated in the rough endoplasmic reticulum (RER) and transported to the Golgi complex. In the RER and Golgi complex, the SARS-CoV-2 glycoprotein is subjected to co-translational and post-translational processing, including signal peptide removal, trimerization, extensive glycosylation and subunit cleavage. The N protein is subsequently associated with the positive sense genomic RNA to become a nucleoprotein complex (nucleocapsid), which together with S, M, and E proteins as well as other viral proteins, is further assembled and followed by budding into the lumen of the ER-Golgi intermediate compartment (ERGIC) to form mature virions. Finally, the mature virions are released from the host cell, waiting for a new life cycle to start. This figure is adapted from the template in BioRender (https://biorender.com/).

The SARS-CoV-2 S glycoprotein is synthesized as a 1273-amino acid polyprotein precursor on the rough endoplasmic reticulum (RER) (**Figure 1**) (13). The unprocessed precursor harbors an endoplasmic reticulum (ER) signal sequence located at the N terminus, which targets the S glycoprotein to the RER membrane and is removed by cellular signal peptidases in the lumen of the ER (14, 15). A single stop-transfer, membrane-spanning sequence located at the C terminus of the S protein prevents it from being fully released into the lumen of the ER and subsequent secretion from the infected cell (16, 17). Co-translationally, N-linked, high-mannose oligosaccharide side chains are added during synthesis (18, 19). Shortly after synthesis, the S glycoprotein monomers trimerize, which might be thought to facilitate the transport from the ER to the Golgi complex. Once in the Golgi complex, most of the high-mannose oligosaccharide side chains are modified to more complex forms (20, 21), and O-linked oligosaccharide side chains are also added (22, 23).

In the trans-Golgi network, the SARS-CoV-2 S glycoprotein is proteolytically cleaved by cellular furin or furin-like proteases at the S1/S2 cleavage site, comprising multiple arginine residues that are not found in the closely related SARS-CoV (24, 25). Cleavage at the S1/S2 site yields a surface subunit S1, which attaches the virus to the host cell surface receptor, and a transmembrane subunit S2, which mediates the fusion of viral and host cell membranes (10). The S1 and S2 subunits remain associated through noncovalent interactions in a metastable prefusion state (11). Furin-like cleavage is essential for the S-protein mediated cell-cell fusion and viral infectivity, and is required for efficient SARS-CoV-2 infection of human lung cells (24) and airway epithelial cells (26).

Following cleavage, an ER retrieval signal (ERRS) consisting of a conserved KxHxx motif (27) located at the extreme C terminus ensures that the mature SARS-CoV-2 S protein accumulates near the ER-Golgi intermediate compartment (ERGIC) (27, 28), where driven by interactions with another structural protein, the membrane (M) protein, the S protein participates in virus particle assembly and is incorporated into virus envelope (**Figure 1**) (29, 30). Besides, a fraction of mature SARS-CoV-2 S proteins travel through the secretory pathway to the plasma membrane, where they can mediate fusion of infected with uninfected cells to form multinucleated giant cells (syncytia) (24, 31). This may allow direct spreading of the virus between cells and potentially alter the virulence of SARS-CoV-2 (24).

Notably, a deletion of ~20 amino acid containing the ERRS from the cytoplasmic tail of the SARS-CoV-2 S protein has been shown to increase the infectivity of single-cycle vesicular stomatitis virus (VSV)-S pseudotypes (9) and replication-competent recombinant VSVs bearing the S glycoprotein (32, 33), which likely could be translated to single-cycle human immunodeficiency virus (HIV)-S or other retrovirus-S pseudotypes straightforward (33). Presumably, this deletion may enhance the cell surface expression of the SARS-CoV-2 S glycoprotein (32), thereby facilitating the S protein incorporation into pseudovirions and replication-competent virions.

## SARS-CoV-2 S Protein Structure and Function 

**Figure 2:**
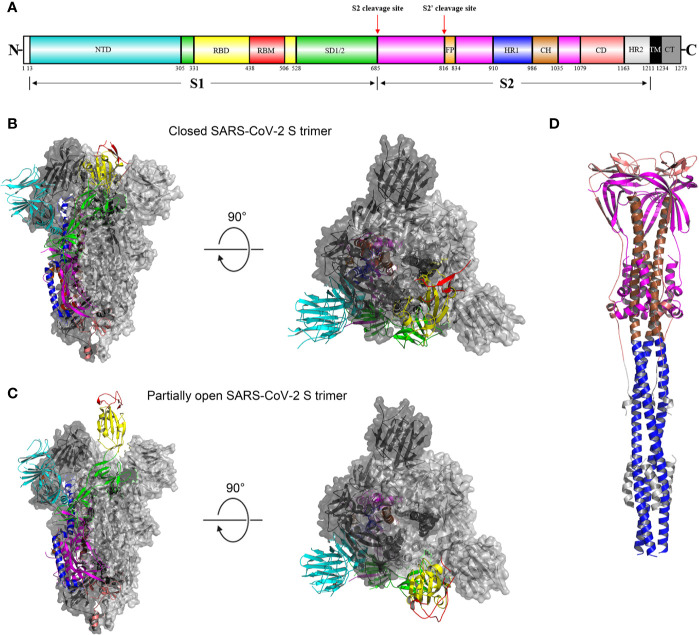
Overall structures of the SARS-CoV-2 S glycoprotein trimer in different conformations. **(A)** Schematic representation of the domain arrangement of the SARS-CoV-2 S protein precursor. SS, signal peptide; NTD: N-terminal domain; RBD: receptor-binding domain; RBM: receptor-binding motif; SD1/2: subdomain 1 and 2; FP, fusion peptide; HR1, heptad repeat 1; CH, central helix; CD, connector domain; HR2, heptad repeat 2; TM, transmembrane domain; CT, cytoplasmic tail. Arrows denote protease cleavage sites. **(B)** Side and top views of the prefusion structure of the SARS-CoV-2 S ectodomain trimer with all three RBDs in the down conformation (PDB ID: 6VXX). One protomer is shown in ribbon representation colored corresponding to the schematic in **(A)**, a second protomer in light gray surface representation, and the third protomer in dark gray surface representation. **(C)** is identical to **(B)** except that a single RBD assumes the up conformation and is shown in ribbon representation (PDB ID: 6VYB). **(D)** Overall structure of the SARS-CoV-2 S2 trimer in the postfusion conformation is shown in ribbon representation colored corresponding to the schematic in **(A)** (PDB ID: 6XRA). The glycans were omitted for clarity.

As mentioned above, the SARS-CoV-2 S glycoprotein plays pivotal roles in viral infection and pathogenesis. Mature S glycoprotein on the viral surface is a heavily glycosylated trimer, each protomer of which is composed of 1260 amino acids (residues 14-1273) (**Figure 2A**). The surface subunit S1 is composed of 672 amino acids (residues 14–685) and organized into four domains: an N-terminal domain (NTD), a C-terminal domain (CTD, also known as the receptor-binding domain, RBD), and two subdomains (SD1 and SD2) (**Figure 2A**) (34). The transmembrane S2 subunit is composed of 588 amino acids (residues 686-1273) and contains an N-terminal hydrophobic fusion peptide (FP), two heptad repeats (HR1 and HR2), a transmembrane domain (TM), and a cytoplasmic tail (CT), arranged as FP-HR1-HR2-TM-CT (**Figure 2A**) (34).

As a typical class I viral fusion protein (35), the SARS-CoV-2 S glycoprotein shares common structural, topological and mechanistic features with other class I fusion proteins, including HIV envelope (Env) glycoprotein and influenza virus haemagglutinin (HA) (36–38). Like other class I viral fusion proteins, the SARS-CoV-2 S glycoprotein is also a conformational machine that mediates viral entry by rearranging from a metastable unliganded state, through a pre-hairpin intermediate state, to a stable postfusion state (38, 39). Since the first genome sequence of SARS-CoV-2 became publicly available (40), a number of structures have been determined for the SARS-CoV-2 S glycoprotein trimer fragments in both the prefusion and postfusion states (**Figures 2B–D**) (11, 34, 41).

The overall architecture of the prefusion SARS-CoV-2 S ectodomain stabilized by two consecutive proline mutations in two conformations determined by single particle cryo-electron microscopy (cryo-EM) is a ~160 Å long trimer with a triangular cross-section, with the S1 subunit adopting a “V” shape contributing to the overall triangular appearance and the S2 subunit forming the stalk (**Figures 2B, C**) (11, 34). The structural difference between these two conformations only lies in the position of one of the three S1 RBDs (**Figures 2B, C**) (11). When all three RBDs are in the “down” position, the resulting S ectodomain trimer assumes a closed conformation, in which the receptor-binding surface of the S1 RBD is buried at the interface between protomers and cannot be accessible by its receptor (**Figure 2B**) (11). The S ectodomain trimer with one single RBD in the “up” position assumes a partially open conformation and represents the functional state, as the receptor-binding surface of the “up” RBD can be fully exposed (**Figure 2C**) (11, 34). The structural information provides a blueprint for structure-based design of vaccine immunogens and entry inhibitors of SARS-CoV-2.

In the closed SARS-CoV-2 S ectodomain trimer, inter-protomer interactions occur through the S1 CTD packed against the other two S1 CTDs and one NTD from an adjacent protomer because of domain swapping and through S2, primarily between helical interactions formed by the upstream and central helices from each subunit around the trimer axis (**Figure 2B**) (11). The S1 subunits rest above the S2 trimer, stabilizing the later in the prefusion conformation (**Figure 2B**) (11). When the S ectodomain trimer adopts a partially open conformation, the RBD in the “up” position will abolish the contacts with the S2 subunit of an adjacent protomer, destabilizing the partially open conformation (**Figure 2C**) (11, 34). This will be beneficial to the dissociation of the S1 subunit and facilitate conformational rearrangements that the S2 trimer undergoes to mediate viral entry.

Prefusion structures of human coronavirus HKU1 (HCoV-HKU1) and mouse hepatitis virus S protein ectodomains without two consecutive proline mutations reveal only fully closed conformation (37, 42), similar to that observed for a full-length, wild-type prefusion form of the SARS-CoV-2 S glycoprotein (41). Notably, it is well established that trimeric prefusion HIV-1 Env primarily resides in a closed configuration that is conformationally masked to evade antibody-mediated neutralization (43, 44) and can spontaneously sample a transient, functional configuration (45). It can thus be speculated that native CoV S glycoproteins on mature and infectious virions share a similar conformational masking feature (46), concealing the receptor-binding surface (for those utilizing CTDs as RBDs) (**Figure 2C**), which is further discussed below.

Several lines of research have established that angiotensin-converting enzyme 2 (ACE2) is an entry receptor for SARS-CoV-2 (47–49). Detailed interactions between the SARS-CoV-2 RBD and its receptor ACE have been revealed by several structures of ACE2 in complex with RBD (50–53). Structurally, RBD consists of two subdomains: a core and an external subdomain (51, 52). An extended loop (residues 438-506), which lies on one edge of the core subdomain, presents a gently concave surface to cradle the N-terminal helix (α1) of ACE2. Analysis of the interface between the SARS-CoV-2 RBD and ACE2 reveals that a total of 17 residues in RBD are in contact with 20 amino acids in ACE2, forming a network of hydrophilic interactions that are suggested to predominate the virus-receptor engagement (51). Outside this extended loop, residue Lys417 located in helix α3 of the core subdomain, was shown to form ionic interactions with Asp30 of ACE2. As the extended loop contains almost all the amino acids of the SARS-CoV-2 RBD that contact ACE2, it is referred to as the receptor-binding motif (RBM) (51).

It has been proposed that inhibiting the interaction between RBD and ACE2 might be useful in treating SARS-CoV-2 infection. Recombinant soluble ACE2 (54) and ACE2-Fc (55, 56) have been shown to have potential applications in the prevention and treatment of SARS-CoV-2 infection *in vitro*. As the interaction between the RBD and ACE2 is extensive, small molecules probably cannot be used as entry inhibitors to effectively block the virus entry by targeting the interaction interface. However, peptides would be able to engage most of the residues belonging to RBM (57). A pioneering study demonstrated that a 23-amino acid peptide (residues 21-43), derived from the N-terminal helix (α1) of ACE2, specifically associates with the SARS-CoV-2 RBD with low nanomolar affinity and disables receptor interactions (57), representing a promising strategy for preventing the virus from invading human cells. In another study, a 65-amino acid peptide (residues 19-83), derived from the N-terminal back-to-back helices (α1 and 2) and composed of most of the residues of ACE2 that mediate interactions with the S protein, shows a similar but probably more potent inhibitory effect (58).

The formation of a trimer-of-hairpins structure (also known as six-helix bundle) comprising HR1 and HR2 in the postfusion conformation is a unifying feature of class I viral fusion proteins (37). The crystal structure of a protein construct in which SARS-CoV-2 HR1 and HR2 were connected by a six-residue hydrophilic flexible linker was determined to be a canonical six-helix bundle structure with a rod-like shape ∼115 Å in length and ∼25 Å in diameter (59). Three HR1 helices form a parallel central coiled-coil with three HR2 helices packing in an oblique, antiparallel manner against deep hydrophobic grooves on the surface of the central coiled-coil (59). Notably, when a full-length S protein construct bearing the native furin-like cleavage site was transiently expressed by Expi293F cells, the purified S proteins contained the dissociated S2 trimer in the postfusion conformation (41). The cryo-EM structure of this trimeric postfusion S2 shows that the central helix (CH) extended regular helices from the central coiled-coil, oriented toward target cells (**Figure 2D**) (41), which forms the longest central triple helical coiled-coil (~180Å) among all known class I transmembrane subunit structures.

The SARS-CoV-2 S trimer in the pre-hairpin intermediate state is very unstable and is just transiently present *in vivo* after triggering by ACE2 engagement, stymieing structural characterization of the S protein in this state (60). However, although this fusion-intermediate phase is very short, it is enough for inhibitory peptides to associate with the pre-hairpin intermediate and block the six-helix bundle formation (39). Furthermore, it has already been shown that the HR1 regions in various human CoVs are highly conserved (61), and therefore could serve as an attractive target for the design and development of potent and broad-spectrum inhibitors of pan-CoVs, including SARS-CoV-2. A highly potent pan-coronavirus fusion inhibitor, EK1C4, has been reported to have good prophylactic and therapeutic potential against SARS-CoV-2 infection (59).

## Glycan Shield of the SARS-CoV-2 S Glycoprotein

As mentioned earlier, the SARS-CoV-2 S proteins are heavily decorated by heterogeneous N-linked glycans projecting from the S trimer surface. The SARS-CoV-2 S sequence encodes up to 22 N-linked glycan sequons per protomer, which likely plays an important role in protein folding (19) and host immune evasion as a glycan shield (62). Of the 22 potential N-linked glycosylation sites on the S protein, 14 were identified to be predominantly occupied by processed, complex-type glycans (63). The remaining eight sites were found to be dominated by oligomannose-type glycans, which are divergent from those founded on host glycoproteins (63). Although glycosylation sites (N165, N234, N343) proximal to the receptor-binding sites on the SARS-CoV-2 S protein can be observed, ACE2 bound to the glycosylated and deglycosylated S ectodomains with nearly identical affinity (1.7 nM vs 1.5 nM) determined by a biolayer interferometry binding assay (64). This observation suggests that the high binding affinity between the SARS-CoV-2 S protein and ACE2 does not depend on the S protein glycosylation.

When the site-specific N-linked glycans are mapped onto the prefusion structure of the SARS-CoV-2 S ectodomain (63), the resulting model exhibited substantially higher levels of glycan-free surface than that revealed by structures of fully glycosylated, trimeric HIV-1 Env ectodomains (65, 66). This suggests that the SARS-CoV-2 S protein is covered by a less dense and less effective glycan shield compared to viral glycoproteins from HIV-1 (36, 66) and Lassa virus (67), which may be beneficial for the induction of humoral immunity and could be good news for a SARS-CoV-2 vaccine (68).

Notably, it has been shown that multiple major viral surface antigens have neutralizing epitopes that are partly or even exclusively composed of carbohydrate moieties (69, 70), exemplified by the HIV-1 Env spike, which could be recognized by a large number of carbohydrate-binding antibodies, including 2G12, PG9, PG16, CH04, PGT121, PGT128, PGT135, and PGT145 (70, 71). In the case of SARS-CoV-2, more recently a potent neutralizing antibody against both SARS-CoV and SARS-CoV-2, S309, has been shown to recognize a highly conserved glycan-containing RBD epitope (72). These observations suggest that carbohydrate moieties could be immunogenic and highlight the need for immunogens to display the glycans important for the recognition of neutralizing antibodies (73); in support of this, specific N-linked glycans on Hemagglutinin has been shown to be essential for the elicitation of broadly neutralizing antibodies against Influenza (74). Accordingly, there has been mounting interest in exploring the potential of immunogenic glycan moieties as vaccine candidates against multiple viruses, including SARS-CoV-2 (75, 76).

## SARS-CoV-2 S Glycoprotein-Mediated Membrane Fusion

Membrane fusion and viral entry of SARS-CoV-2 is initiated by binding of RBD in the viral S glycoprotein transiently sampling the functional conformation to ACE2 on the surface of target cells (**Figure 1**) (10). After receptor engagement at the plasma membrane or ensuing virus endocytosis by the host cell (8), a second cleavage (S2′ cleavage site) is generated, which is mediated by a cellular serine protease TMPRSS2 (48) or endosomal cysteine proteases cathepsins B and L (10) (**Figure 1**). Protease cleavage at S2′ site frees the fusion peptide from the new S2 N-terminal region, further destabilizes the SARS-CoV-2 S glycoprotein and may initiate S2-mediated membrane fusion cascade. Following the second cleavage, the fusion peptide at the N terminus of the S2 trimer is inserted into the host membrane (8), forming the pre-hairpin intermediate state (39). Since the pre-hairpin intermediate state is extremely unstable, the S2 fusion protein is refolded quickly and irreversibly into the stable postfusion state (39, 77). These large conformational rearrangements pull the viral and host cell membrane into close proximity, leading ultimately to the membrane fusion (8, 39).

## Insights Into the Design and Development of S Protein-Based Vaccines

Since SARS-CoV-2 was identified as the causative agent of COVID-19, and its first genome sequence was released immediately and freely by a Chinese research group (40), SARS-CoV-2 vaccine candidates based on various vaccine platforms, such as inactivated or live attenuated vaccines, DNA and mRNA vaccines, viral vector-based vaccines, and recombinant protein-based vaccines, have been developed (12, 78). Most of these vaccine strategies are based on the full-length S glycoprotein, the major viral surface antigen (12). When a vaccine strategy requires that the SARS-CoV-2 S protein be recombinantly expressed in the human body, the ERRS should be omitted to enhance the cell surface expression level of the resulting protein.

Theoretically, the native HIV-1 Env trimer present on the surface of intact virions is thought to be a most ideal immunogen (60), as most of the neutralizing antibodies thus far described could recognize and bind to the prefusion form of trimeric HIV-1 Env, although it is with great difficulty that such neutralizing antibodies against this glycan-covered, sequence-variable native form are induced (36). For SARS-CoV-2, different lines of research have shown that convalescent sera from SARS-CoV and SARS-CoV-2 patients showed no or limited cross-neutralization activity against these two viruses by pseudotyped and authentic viral infection assays, despite significant cross-reactivity in binding to the S glycoproteins of both viruses (9, 79–81). Similar results were also observed in infected or immunized animals (48, 79, 81). Together with the finding that although the SARS-CoV-2 S protein shares a high degree of amino acid sequence identity with that of SARS-CoV (~76% overall), the RBM is less conserved (~47% identity) than any other functional region or domain (82), it can thus been surmised that the RBM has the most immunodominant neutralizing epitope(s) of the whole S protein, capable of readily eliciting strong neutralizing antibody responses. However, the native trimeric SARS-CoV-2 S protein could conceal each of its immunodominant RBMs by adopting the closed conformation (41, 83). Therefore, SARS-CoV-2 evades immune surveillance also through conformational masking, which is well-documented for HIV-1 (43, 44); while at the same time, the S protein could transiently sample the functional state to engage ACE2, consistent with the notion that the fusion glycoprotein of highly pathogenic viruses have evolved to perform its functions while evading host neutralizing antibody responses.

Another concern for vaccine candidates based on the full-length S glycoprotein of SARS-CoV-2 is raised by the observation that the S1 subunit could spontaneously dissociate from the S glycoprotein probably as a trimer that still assumes the RBD closed conformation, leaving only the postfusion S2 trimer (41). The resulting S1 and S2 subunits might expose immunodominant, nonneutralizing epitopes that are utilized by SARS-CoV-2 to serve as decoys to distract the host immune system, inducing a large proportion of ineffective antibody responses, as documented for HIV-1 (60) and respiratory syncytial virus (RSV) (84).

It should be noted that although vaccine candidates based on the full-length S protein of the closely related SARS-CoV could elicit neutralizing antibody responses against infection of SARS-CoV, they may also induce harmful immune responses, including liver damage of the vaccinated animals, infection of human immune cells by SARS-CoV, and antibody-dependent enhancement of SARS-CoV infection (85–89). Therefore, although the S proteins of both SARS-CoV and SARS-CoV-2 are thought to be promising vaccine immunogens for generating protective immunity, optimizing antigen design is critical to ensure an optimal immune response through exposing more neutralizing epitopes and displaying fewer potentially weakly or non-neutralizing epitopes (90). Vaccines containing or expressing the full-length S protein or its soluble ectodomain form should thus be engineered to sample a RBD(s) “up” conformation while the rest is still kept in the prefusion state (91, 92).

Apart from recombinant, soluble, stabilized ectodomains that are engineered to expose the immunodominant RBD by adapting the RBD(s) “up” conformation, RBD proteins of SARS-CoV and SARS-CoV-2 have also been widely used as recombinant protein-based vaccines (85, 93–95). The RBD of SARS-CoV is highly immunogenic (96, 97) and is targeted by most of the neutralizing monoclonal antibodies that have been characterized (98). Based on the observation that a 193-amino acid fragment (residues 318-510) was previously identified to be the minimal RBD region of SARS-CoV (99), a corresponding 194-amino acid fragment (residues 331-524) can be readily selected as the minimal RBD region of SARS-CoV-2 and has already been characterized (100). This minimal form of RBDs of both viruses could serve as a vaccine candidate (100).

However, a conserved cysteine residue is located immediately upstream of the minimal RBD fragments of both viruses and always forms a disulfide bond in nearly all published structures containing this residue (101, 102); this is also the case for Middle East respiratory syndrome coronavirus (MERS-CoV) (103, 104) and HCoV-HKU1 (37), consistent with the observation that all RBDs of these viruses share a conserved structural core. The disulfide bond contributes to stabilization of the RBD structure and likely modulates the protein immunogenicity. This notion is consistent with the observation that mice immunized with a longer form of the SARS-CoV RBD (residues 318-536) produced a higher titer of neutralizing antibodies compared with mice immunized with the minimal RBD region (residues 318-510) (105). Therefore, when each of the minimal RBD fragments of SARS-CoV and SARS-CoV-2 is used as vaccine candidates, the critical cysteine residue should not be ignored and thus should be included (106).

Besides the RBD, which has been shown to a major target for human neutralizing antibody responses (107), the NTD was recently identified to be a new vulnerable site of the SARS-CoV-2 S protein for antibody neutralizing and therefore could also serve as a recombinant protein-based vaccine (108–110). As expected, NTD-specific neutralizing antibodies could target the S protein in both closed and open conformations (108). In addition, the apparent accessibility of the fusion peptide and HR1 region in published structures of the SARS-CoV-2 S ectodomain trimer as well as their high sequence conservation among CoVs suggests that they would be good immunogen candidates for epitope-focused vaccine design aimed at raising broadly CoV neutralizing antibodies (46). The epitope-focused vaccine design has proven to be successful in generating neutralizing antibodies against RSV fusion glycoprotein (111). However, neutralizing antibodies targeted against these two regions still need to be isolated in infected individuals to support this notion.

Unlike wild-type full-length S protein of SARS-CoV-2, the above monomeric fragments do not induce any infection-enhancing antibodies or harmful immune or inflammatory responses (106, 112), all of which could be potentially avoided through structure-based immunogen design to improve immunogenicity (113, 114). However, wide-type full-length or soluble ectodomain form of the SARS-CoV-2 S protein could trigger stronger cellular immune responses (115), which have been demonstrated to play an important role in controlling diseases caused by CoVs (116, 117), including SARS-CoV-2 (118), and are probably also an important determinant of effective vaccines against SARS-CoV-2 (115, 119). Additionally, when more than one RBD of the S protein trimer is engineered to be locked in the “up” conformation (120, 121), the antigenicity and immunogenicity of the resulting RBDs would be significantly enhanced compared to monomeric RBD form (97, 122). Moreover, improved protection is likely to be achieved when vaccinated with full-length or soluble ectodomain form of the SARS-CoV-2 S protein in that both forms can elicit neutralizing antibodies directed against non-RBD sites, as observed for MERS-CoV (123).

Genetic variation has been used by many viruses that have RNA genomes (124), including HIV and influenza, as a mechanism to avoid antibody-mediated immunity, and is partially responsible for the great difficulty in developing effective and durable vaccines against these viruses (36). As an RNA virus, however, SARS-CoV-2 has a very low mutation rate overall (125) likely because CoVs have a genetic proofreading mechanism (126). All reported variations occurred in the SARS-CoV-2 S glycoprotein have a prevalence of no more than 1% (127), with an exception of D614G, which has become the most prevalent genotype in the global COVID-19 pandemic (127). Fortunately, although the D614G mutation of the SARS-CoV-2 S protein has been shown to enhance viral infectivity (128–130), until now there is no evidence that infection with SARS-CoV-2 carrying the G614 mutant will be associated with disease severity (127, 131). Furthermore, assays using both monoclonal and polyclonal antibodies generated from individuals naturally infected with D614- or G614-carrying viruses demonstrated that the D614G mutation retains or even increases viral susceptibility to neutralization (127, 130, 132, 133). This suggests that the D614G mutant maintains or favors an open, functional conformational state (134).

Although at an extremely low frequency, natural variations, including L452R A475V, V483A, and F490L that render the S glycoprotein resistant to certain neutralizing antibodies targeting the RBD, emerged under no selection pressure exerted by approved vaccines or neutralizing antibodies or entry inhibitors (127, 132). However, it has been shown that SARS-CoV-2 escape mutants could be easily selected and quickly amplified under the selection pressure of single antibody treatment (135). These observations suggest that a combination of at least two neutralizing antibodies that recognize and bind to distinct and non-overlapping epitopes on the SARS-CoV-2 S glycoprotein (e.g., RBD and NTD, as well as HR and glycan) is required to restrict the possible occurrence of viral escape mutants and potential subsequent loss of single antibody-mediated neutralization (135–138). When these observations are taken into consideration for vaccine design and development, an ideal SARS-CoV-2 immunogen should contain as many exposed neutralizing epitopes as possible, although the RBD also possesses extra epitope(s) besides the epitope in the RBM region (72, 139–141).

## Concluding Remarks and Prospects

SARS-CoV-2 is a highly contagious pathogen that continues to spread quickly around the globe, causing COVID-19 to be one of the worst pandemics in recorded history. A safe and efficacious vaccine represents one of the best ways to reduce or eliminate the COVID-19 pandemic (142). Unfortunately, no vaccines for any of the known human CoVs have been licensed (143, 144), although several potential SARS-CoV and MERS-CoV vaccines have advanced into human clinical trials for years (117, 145), suggesting the development of effective vaccines against human CoVs has always been challenging. However, it has been shown that both SARS-CoV and SARS-CoV-2 could readily induce neutralizing antibodies following natural infection or immunization (146–149). Moreover, a growing number of neutralizing monoclonal antibodies targeting the SARS-CoV-2 S glycoprotein with high potency have been isolated from plenty of convalescent donors (33) as well as humanized mice (136, 141), some of which have been shown to afford protection against SARS-CoV-2 challenge in animal models. It thus seems that vaccine candidates designed to elicit such neutralizing antibodies are feasible. It is widely accepted that the S protein of SARS-CoV-2 is a most promising immunogen for producing protective immunity (150). However, it is likely that the S protein has evolved to perform its functions while evading host neutralizing antibody responses and thus should be engineered to ensure an optimal immune response (151, 152). The immunogen design strategies described in this review based on the wealth of the SARS-CoV-2 S glycoprotein research related to its biosynthesis, structure, function, antigenicity as well as immunogenicity will likely contribute to the ultimate success of safe and efficacious vaccines against SARS-CoV-2/COVID-19.

## Author Contributions

All authors listed have made a substantial, direct, and intellectual contribution to the work, and approved it for publication.

## Funding 

Our research was supported by the Natural Science Foundation of Henan Province (Grant No.182300410327), the National Natural Science Foundation of China (Grant No. 81871312 and 81701546), and by the 111 Project (No. D20036).

## Conflict of Interest

The authors declare that the research was conducted in the absence of any commercial or financial relationships that could be construed as a potential conflict of interest.
